# Diagnostic utility of transcranial magnetic stimulation for neurodegenerative disease: a critical review

**DOI:** 10.1590/1980-5764-DN-2023-0048

**Published:** 2024-01-05

**Authors:** Javier Moreno-Roco, Lucía del Valle, Daniel Jiménez, Ignacio Acosta, José Luis Castillo, Thanuja Dharmadasa, Matthew C. Kiernan, José Manuel Matamala

**Affiliations:** 1 Universidad de Chile, Facultad de Medicina, Laboratorio de Neurología y Neurofisiología Traslacional, Santiago, Chile; 2 Universidad de Chile, Facultad de Medicina, Centro de Investigación Clínica Avanzado (CICA) Oriente, Santiago, Chile.; 3 Universidad de Chile, Facultad de Medicina, Departamento de Ciencias Neurológicas Oriente, Santiago, Chile.; 4 Universidad de Chile, Facultad de Medicina, Departamento de Neurociencias, Santiago, Chile.; 5 Hospital del Salvador, Servicio de Neurología, Santiago, Chile.; 6 University of Melbourne, The Florey Institute of Neuroscience and Mental Health, Parkville, Victoria, Australia.; 7 The Royal Melbourne Hospital, Department of Neurology, Parkville, Victoria, Australia.; 8 University of Sydney, Brain and Mind Centre, Sydney, Australia.; 9 Royal Prince Alfred Hospital, Department of Neurology, Sydney, AustraliaArgento; 10 Universidad de Chile, Facultad de Medicina, Instituto de Neurociencia Biomédica (BNI), Santiago, Chile.

**Keywords:** Transcranial Magnetic Stimulation, Neurophysiology, Biomarkers, Neurodegenerative Diseases, Amyotrophic Lateral Sclerosis, Estimulação Magnética Transcraniana, Neurofisiologia, Biomarcadores, Doenças Neurodegenerativas, Esclerose Amiotrófica Lateral

## Abstract

Neurodegenerative diseases pose significant challenges due to their impact on brain structure, function, and cognition. As life expectancy rises, the prevalence of these disorders is rapidly increasing, resulting in substantial personal, familial, and societal burdens. Efforts have been made to optimize the diagnostic and therapeutic processes, primarily focusing on clinical, cognitive, and imaging characterization. However, the emergence of non-invasive brain stimulation techniques, specifically transcranial magnetic stimulation (TMS), offers unique functional insights and diagnostic potential. TMS allows direct evaluation of brain function, providing valuable information inaccessible through other methods. This review aims to summarize the current and potential diagnostic utility of TMS in investigating neurodegenerative diseases, highlighting its relevance to the field of cognitive neuroscience. The findings presented herein contribute to the growing body of research focused on improving our understanding and management of these debilitating conditions, particularly in regions with limited resources and a pressing need for innovative approaches.

## RESUMO.

As doenças neurodegenerativas representam desafio significativo por seu impacto na estrutura cerebral, função e cognição. À medida que a expectativa de vida aumenta, a prevalência dessas doenças cresce rapidamente, resultando em substanciais encargos pessoais, familiares e sociais. Esforços têm sido feitos para otimizar os processos diagnósticos e terapêuticos, com foco principal na caracterização clínica, cognitiva e de imagem. No entanto, o surgimento de técnicas de estimulação cerebral não invasivas, especificamente a estimulação magnética transcraniana (EMT), oferece compreensão funcional e potencial diagnóstico únicos. A TMS permite a avaliação direta da função cerebral, fornecendo informações valiosas inacessíveis por outros métodos. Esta revisão teve como objetivo resumir a utilidade diagnóstica atual e potencial da EMT na investigação de doenças neurodegenerativas, destacando sua relevância para o campo da neurociência cognitiva. As conclusões aqui apresentadas contribuem para o crescente corpo de investigação centrado na melhoria da nossa compreensão e gestão dessas condições debilitantes, particularmente em regiões com recursos limitados e necessidade premente de abordagens inovadoras.

## INTRODUCTION

Neurodegenerative diseases represent brain alterations characterized by the progressive damage of selectively vulnerable populations of neurons, resulting in morphological and functional disruption of specific areas in the central nervous system (CNS). They can be primarily classified according to specific clinical symptoms/signs (*e.g.*, in the case of dementia, parkinsonism, motor neuron disease [MND] / amyotrophic lateral sclerosis [ALS]), anatomic distribution, molecular abnormalities, or neuropathological findings, that have distinguishable cognitive, imaging, and neurophysiological features^1^.

Dementias are the most common neurodegenerative disorders and are defined as a clinical syndrome characterized by progressive cognitive decline that interferes with the ability to function independently^2^. The most common etiologies that cause dementia are Alzheimer's disease (AD), Lewy body disease (LBD), and frontotemporal dementia (FTD). Parkinson's disease (PD) is the most common cause of parkinsonism and the second most common neurodegenerative disorder, characterized by progressive extra-motor symptoms like rigidity, bradykinesia, and tremor. Finally, MND is a rare group characterized by progressive loss of upper (UMN) and lower motor neurons (LMN). ALS is the most common subtype of MND, accounting for 80-90% of all MND cases^3^.

As the risk of developing neurodegeneration increases with age, the incidence and prevalence of these conditions will rapidly rise over the next decades in this context of an aging population. According to the United Nations, the number of persons over 65 years of age will more than double by 2050 worldwide^4^, which would result in the prevalence of neurodegenerative diseases doubling or even tripling by that decade. For example, the global number of people living with dementia is projected to grow by more than double every 20 years^5^, and a sustained increase in PD's prevalence and incidence has been observed since 2019 in most regions of the world^6^.

Over recent years, biomarkers have been developed to represent measurable indicators of a biological state or pathological condition^7^, including genetic, neuroimaging, and biofluid approaches^8^. In addition, non-invasive techniques have been explored as biomarkers for neurodegeneration, since they complement the study of these pathologies with functional variables that provide valuable information about brain activity in a spatial and temporal range that is not accessible through other methods, representing measures of cortical excitability, plasticity, and network connectivity.

An important limitation of the study of these diseases and development of new potential treatments lies in the lack of research in Latin American and Caribbean (LAC) countries, and as such:

- the real impact of these pathologies at the epidemiological level is unknown;
- the relevant clinical, molecular, and genetic differences that could be grouped in these regions remain undefined.

Considering these challenges, this review will analyze the utility of transcranial magnetic stimulation (TMS) for diagnosis.

## TRANSCRANIAL MAGNETIC STIMULATION

Following the introduction of the first non-invasive brain stimulation device by Merton and Morton in 1980^9^, transcranial electrical stimulation (TES), clinical neurophysiology, and brain stimulation techniques have undergone a radical turn. This first tool required high-voltage electric stimuli on the scalp to evaluate excitability properties on CNS fibers but also produced significant pain. TMS was thus developed as an alternative non-invasive brain stimulation device to enable activation of certain areas of the CNS. First presented by Barker and colleagues in 1985^10^, this method contrasted TES by using principles of electromagnetic induction. As described by Michael Faraday almost 50 years before the invention of TMS, a high-intensity electric pulse sent through a wire loop can generate a magnetic field that is perpendicular to the plane of the loop, but in opposite direction to the original current. Using this principle, short- duration current pulses (<1 ms) pass through a copper coil, generating a magnetic field that reaches around 2 tesla and lasts 100 ms. This magnetic field passes through the soft tissues and the skull without being attenuated and can induce a secondary electric field in the brain cortex^11^. The induced electric field can trans synaptically activate pyramidal cells^12^ and, consequently, trigger action potentials in the targeted cortical areas. To date, several types of stimulators have been developed that vary according to research purposes, as well as a variety of coil types which determine the area and depth of induction.

TMS is a broad and versatile tool to evaluate different neurophysiological variables according to the stimulation protocols that are applied. Protocols differ in temporality, intensity, frequency, and number of stimuli that are given. Applied on the primary motor cortex (M1), most of the protocols evaluate excitability through interrogation of the corticomotoneuronal or corticobulbar pathways, although it has also been used to emulate paradigms of neuronal plasticity. Table 1 summarizes the evidence available to date related to physiological relevance and neurotransmitters involved in single- pulse TMS and paired- pulse TMS and neuroplasticity protocols that are discussed below. The potential diagnostic utility of TMS-EEG (electroencephalography) is not discussed and the reader is referred to dedicated reviews on this topic^13^.

**Table 1:** Main protocols to assess cortical excitability and neuroplasticity through transcranial magnetic stimulation and its related physiological circuits and neurotransmitters described in the literature.

Protocols		Proposed physiological mechanisms	Neurotransmitters involved
Single-Pulse TMS	MEP	Summation of corticospinal volleys of direct and indirect waves onto corticospinal neurons. Global excitability	Mainly glutamatergic synapses through corticomotoneuronal system
	RMT	Density of corticomotoneuronal projections and their global excitability	Mainly glutamatergic synapses through corticomotoneuronal system
	CSP	Cortico-subcortical and spinal excitability	Mainly GABA_B_-type receptors
	ISP	Evaluate transcallosal inhibition	Glutamatergic and GABAergic synapses
	CMCT	Excitability of the fastest conducting corticomotoneuronal projections	Mainly glutamatergic synapses through corticomotoneuronal system
Paired-Pulse TMS	SICI	Inhibitory short-interval intracortical circuits	GABA_A_-type receptors mediated
	ICF	Facilitatory short-interval intracortical circuits	Glutamatergic excitatory postsynaptic potentials (NMDA receptor)
	SICF	Facilitatory short-interval intracortical circuits	Facilitatory activity from interneuronal activation resulting in summation of I-waves on corticospinal neurons
	LICI	Inhibitory long-interval intracortical circuits	Mediated in part by GABA_B_-type receptors
	SAI	Motor cortex inhibition induced by short-latency peripheral afferents stimulus	Cholinergic thalamocortical projections on GABA_A_ cortical networks
	LAI	Motor cortex inhibition induced by long-latency peripheral afferents stimulus	Inhibitory interneurons that are shared by LICI
	CBI	Inhibitory dento-thalamo-cortical pathway	GABA_A_ receptors at the end of cerebellothalamocortical pathway
Neuro-plasticity TMS	iTBS	Corticospinal or corticocortical excitability that may reflect LTP-like synaptic effects	Glutamatergic NMDA receptors mediating LTP-like synaptic effects
	cTBS	As iTBS, but may reflect LTD-like synaptic effects	May mediate LTD-like synaptic effects by GABAergic transmission
	HF rTMS	LTP-like synaptic effects	May mediate LTD-like effects through slow increase in ionic calcium concentration
	LF rTMS	LTD-like synaptic effects	May mediate reduce cortical excitability generating later I-waves
	PAS	LTP-like effects through Hebbian STDP	Mainly glutamatergic NMDA receptors mediating LTP-like effects

Abbreviations: TMS, transcranial magnetic stimulation; MEP, motor evoked potential; RMT, resting motor threshold; CSP, cortical silent period; GABA, gamma aminobutyric acid; ISP, ipsilateral silent period; CMCT, central motor conduction time; SICI, short-interval intracortical inhibition; ICF, intracortical facilitation; NMDA, N-methyl-D-aspartate; SICF, short-interval intracortical facilitation; I-waves, indirect waves; LICI, long-interval intracortical inhibition; SAI, short-latency afferent inhibition; LAI, long-latency afferent inhibition; CBI, cerebellar brain inhibition; iTBS, intermittent theta burst stimulation; LTP, long-term potentiation; cTBS, continuous theta burst stimulation; LTD, long-term depression; HF rTMS, high frequency repetitive transcranial magnetic stimulation; LF rTMS, low frequency repetitive transcranial magnetic stimulation; PAS, paired associative stimulation; STDP, spike timing dependent plasticity.

### Neurophysiological evaluation using single-pulse protocols

When applied to M1, single pulse stimulation induces contralateral muscle contraction in a somatotopically organized distribution. The resulting muscular output can be registered through standard surface electrodes as a motor evoked potential (MEP). Some variables can be measured using a single pulse, including motor threshold (MT), central motor conduction time (CMCT), and cortical silent period (CSP), as discussed below.

MT is determined in order to standardize MEP between individuals. It is defined by the International Federation of Clinical Neurophysiology as the minimum intensity required to elicit a MEP amplitude greater than 50 mV (peak-to-peak) in the target muscle on five of ten consecutive stimuli and is noted as a percentage of maximal stimulator output (MSO). When the single pulse is given in a resting condition, this variable is called resting motor threshold (RMT)^14^. Similarly, active motor threshold (AMT) is expressed as the percentage MSO required to elicit an MEP amplitude greater than 200 mV in the target muscle on five of ten consecutive stimuli while the individual maintains a light contraction^14^.

CMCT corresponds to the time elapsed for TMS stimulus to go through the CNS and is calculated by subtracting the peripheral motor conduction time from MEP latency^14^. Finally, when TMS is given during voluntary contraction, a reduction in electromyographic activity follows MEP, which is known as CSP^14^. Conversely, during muscle contraction, a single TMS pulse over ipsilateral M1 can be given to evoke a silent period in the background activity generated by ipsilateral muscle, which is known as the ipsilateral silent period (ISP)^14^.

### Paired-pulse protocols

Paired- pulse TMS protocols are thus employed to assess cortical circuitry more globally, with consideration of these intracortical circuits. These utilize two stimuli delivered in close succession (<200 ms). These pulses are given at the same region or could be applied in different cortical areas to evaluate their functional connectivity. The first (conditioning) pulse modulates the effect of a second (test) pulse, according to the interstimulus interval (ISI) and the intensity of each pulse.

Short-interval intracortical inhibition (SICI) paradigm can quantify inhibitory effect of interneurons in cortical layers II and III of M1. It happens when two TMS stimuli, one subthreshold conditioning stimulus (S1) followed by a suprathreshold test stimulus (S2), are applied over M1 between an ISI of 1 to 7 ms. In healthy subjects and under normal conditions, this results in a reduction in MEP amplitude when compared with that generated from an isolated single- pulse MEP^14^. On the contrary, short-interval intracortical facilitation (SICF) is a facilitatory paradigm that is reproduced when S1 and S2 are set around RMT and are given between 1 and 7 ms^15^. In the same way, if the two pulses are given between an ISI of 10 to 30 ms, an increase in MEP amplitude is typically seen, called intracortical facilitation (ICF).

When two suprathreshold stimuli are given between 50 to 200 ms of ISI, long-interval intracortical inhibition (LICI) is elicited. There is no direct correlation between the degree of SICI and LICI, in fact, LICI has a suppressive effect on SICI, suggesting that both processes are mediated by different neural circuits^16^. Finally, cerebellar connectivity can be evaluated through cerebellar brain inhibition (CBI), giving a cerebellar conditioning stimulus and a M1 test stimulus using a double-coil protocol^14^.

Modification of paired- pulse protocols can also be made based on a sensorimotor cortical integration process^14^. For example, when an electrical stimulation is applied to a mixed nerve such as median or ulnar (conditioning stimulus) before a TMS pulse delivered over the corresponding central area (test stimulus), a reduced MEP amplitude is obtained. If the two stimuli differ by 20 ms, it represents a short-latency afferent inhibition (SAI)^14^, and if the ISI of the two pulses is 200 ms, long-latency afferent inhibition (LAI)^14^.

Interestingly, the application of these protocols has varied in conjunction with the development of techniques in a constant search for better reproducibility and reliability of the results. In this context, it has been seen that the application of constant stimulus and amplitude measurement techniques could generate variability in successive stimuli. Threshold tracking technique was developed to overcome this limitation by modifying the stimulus intensity for a constant (tracked) target amplitude, generally of 0.2mV±20%^17,18^. This is a well-established technique validated not only in healthy subjects but also in neurodegenerative diseases^16^. Importantly, it has been reported to have greater reliability when compared to the constant stimulus method^19^.

### Neurophysiological evaluation using neuroplasticity-like protocols

It is interesting to note that as neurodegeneration causes specific and progressive dysfunction of different neuronal circuits, it could be related to alterations in brain plasticity^20^ and the search for alterations in the neuroplastic properties of the CNS has been a field of development in the past decade. The use of TMS has not only been limited to exploring cortical excitability with single- and paired- pulse protocols, but also other protocols have attempted to produce *in vivo* modulations of plasticity in the cortex through long-term potentiation (LTP)-like or long-term depression (LTD)-like mechanisms that outlasts the duration of the protocol stimulation^14^ and are discussed below. Modulation of cortical plasticity provides an interesting method to search for early changes in neurodegenerative diseases, as in AD^21^ or other disorders where neuronal circuits may be impaired.

Repetitive transcranial magnetic stimulation (rTMS) can be applied in specific patterns to modulate cortical plasticity with a lasting effect, even up to one hour. It has been used to produce bidirectional modulation of plasticity. Some protocols like intermittent theta burst stimulation (iTBS) and high -frequency (≥5Hz) rTMS produce LTP-like phenomena. Conversely, continuous theta burst stimulation (cTBS) and low frequency rTMS (<5 Hz) produce LTD-like effects^14^.

Paired associative stimulation (PAS) causes modulation of plasticity in M1 by spike-timing dependent synaptic plasticity, based on Hebb's theoretical framework^22^. In this protocol, a peripheral electric stimulation is delivered 20 to 25 ms (PAS25) preceding a central TMS pulse over the representation of the specific muscle targeted. MEP amplitude is increased for about 30 to 40 minutes following the protocol, producing LTP-like effects. Conversely, if the ISI between peripheral and central stimuli is 10 ms (PAS10) LTD-like effects are produced in M1^14^.

## DIAGNOSTIC UTILITY OF TMS FOR NEURODEGENERATIVE DISEASES

TMS techniques provide essential measures of pathophysiological processes developed in neurodegeneration and, therefore, could be employed as a diagnostic biomarker in clinical settings and therapeutic clinical trials. The role of TMS was reviewed and discussed here as a diagnostic support in three groups of diseases (*i.e*. ALS, AD/FTD, and PD). Table 2 summarizes the main changes in TMS cortical excitability and neuroplasticity protocols.

**Table 2:** Main patterns reported in cortical excitability and neuroplasticity protocols through transcranial magnetic stimulation for the diagnosis of neurodegenerative diseases.

Protocol	Single- pulse protocols					Paired pulse protocols							Neuroplasticity protocols				
Disease	MEP	RMT	CSP	ISP	CMCT	SICI	ICF	SICF	LICI	SAI	LAI	CBI	iTBS	cTBS	HF TMS	LF TMS	PAS
ALS	↑	↓	↓		↑	↓	*	↑		↓	↓						↓
AD	↑	↓	↓	↑	=	↓	=		↓	↓							↓
FTD	↓	↑	↓		↑	↓	↓		↓	*			*	*			↓
PD	*	*	*	*	*	*	*	*	*	*	↓	↓	↓				*

Abbreviations: MEP, motor evoked potential; RMT, resting motor threshold; CSP, cortical silent period; ISP, ipsilateral silent period; CMCT, central motor conduction time; SICI, short-interval intracortical inhibition; ICF, intracortical facilitation; SICF, short-interval intracortical facilitation; LICI, long-interval intracortical inhibition; SAI, short-latency afferent inhibition; LAI, long-latency afferent inhibition; CBI, cerebellar brain inhibition; iTBS, intermittent theta burst stimulation; cTBS, continuous theta burst stimulation; HF TMS, high frequency repetitive transcranial magnetic stimulation; LF TMS, low frequency repetitive transcranial magnetic stimulation; PAS, paired associative stimulation; ALS, amyotrophic lateral sclerosis; AD, Alzheimer's disease; FTD, frontotemporal dementia; PD, Parkinson's disease; ↑, increase in protocol excitability or plasticity; ↓, the opposite; =, there is no variation in relation to healthy subjects;

* controversial results in the literature; blank cell, no data available in the literature to date.

### Amyotrophic lateral sclerosis

ALS is a progressive and fatal neurodegenerative disease of the central nervous system, characterized by the degeneration of LMN in the brainstem and spinal cord, along with the concurrent loss of UMN^23^. While the primary clinical manifestations of ALS involve spinal and bulbar regions, as well as the involvement of both LMN and UMN, there is growing recognition of the heterogeneous nature of the disease, including its impact on extra-motor brain areas such as cognitive impairment.

From a pathophysiological perspective, ALS appears to be a multi-stage disease, which requires a sequence of 2-to-6 steps that are influenced by genetic mutations^23^. Recent evidence has highlighted a cortical origin of ALS, suggesting that cortical hyperexcitability may play a role in mediating the degeneration of LMN through a transsynaptic glutamatergic excitotoxic process^3^. This implies that cortical dysfunction and aberrant excitatory neurotransmission may contribute to the progression of the disease, extending beyond the traditional focus on motor neuron degeneration.

Diagnosis of ALS is made by identifying concomitant symptoms and signs of UMN and LMN dysfunction, with evidence of disease progression across specific body regions^24^. Without a pathognomonic diagnostic biomarker, clinically based criteria were initially proposed to facilitate diagnosis, with modest sensitivity, especially in early stages of the disease^24^. In 2008, the neurophysiology- based Awaji criteria were developed to reduce diagnostic delays, whereby electromyography (EMG) findings of chronic neurogenic and ongoing neurogenic changes or fasciculations were considered equivalent to LMN signs^24^. Many subsequent studies have shown that using these clinical criteria modestly improves the diagnostic accuracy compared to the revised El Escorial criteria. Diagnostic delays in ALS significantly impact quality of life and management of patients, and delay patients’ inclusion into therapeutic clinical trials. With this problem in mind, a new diagnostic criterion was proposed^24^, simplifying the previous diagnostic categories into a single entity to better reflect clinical practice. A limitation of ALS diagnostic criteria pertains to the clinical assessment of UMN dysfunction^25^. The Gold Coast consensus group recognized developments in novel biomarkers of UMN degeneration, such as TMS. Future validation of these biomarkers might lead to redefined ALS criteria.

To assess the involvement of UMN and the corticomotoneuronal system in ALS, single-pulse TMS studies have provided significant insights. These studies have demonstrated marked hyperexcitability, characterized by a reduction in RMT along with an increase in MEP amplitude (normalized by compound muscle action potential – CMAP) and a decrease in CSP duration. Paired-pulse protocols have also revealed a decrease or absence of SICI as well as an increase in ICF and in SICF^26,27^.

**Figure 1:**
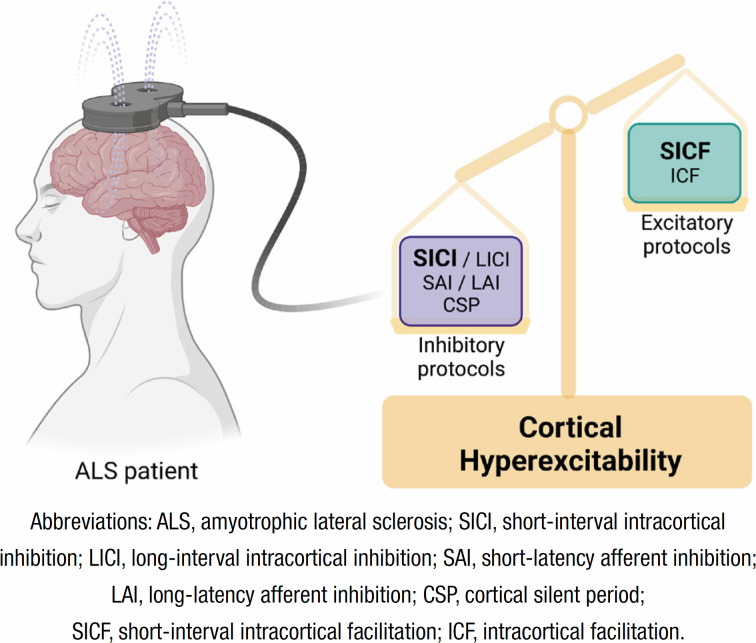
Main alterations reported in cortical excitability protocols through transcranial magnetic stimulation in amyotrophic lateral sclerosis patients. Cortical hyperexcitability phenomena support the changes found. In addition, the most consistent protocols in this disease are highlighted in boldface type, short-interval intracortical inhibition and short-interval intracortical facilitation (created with BioRender.com).

Furthermore, a study examining the relationship of SAI in ALS patients showed a tendency toward reduction, although it was not correlated with cognitive or other neurophysiological variables^28^. However, SICF has been reported to be increased in ALS patients with cognitive impairment and independently associated with cognitive function determined by the Edinburgh Cognitive and Behavioral ALS Screen (ECAS) scale^29^. Similarly, SICI reduction has been reported to be more prominent in ALS patients with worse cognitive performance, indicating even greater hyperexcitability in patients with cognitive decline within the ALS group^29^. In summary, these findings collectively support a state of hyperexcitability in the corticomotoneuronal system, which is likely attributed to the degeneration of inhibitory interneurons and reduced function of GABAA receptors^28^ (Figure 1).

Preclinical studies have shown frequent abnormalities in the cortical interneuron population through reduction of inhibitory currents mediated by GABA receptors in pyramidal neurons, and an alteration in the GABAergic signaling that determines cortical hyperexcitability^30^. In addition, it has recently been seen in animal models that the restoration of intracortical inhibition reduces hyperexcitability in pyramidal neurons, which delays the presentation and progression of the disorder while increasing survival^31^.

Importantly, cortical hyperexcitability is an early property in sporadic as well as familial patients, preceding the clinical presentation in patients with superoxide dismutase-1 (SOD-1) mutation by approximately 3 to 6 months^32^. This phenomenon has also been observed in patients with chromosome 9 open reading frame 72 (C9ORF72) gene expansion^33^, but not in patients expressing homozygous D90A SOD-1 mutation, which have relative preservation of cortical inhibitory mechanisms^34^. These studies indicate that cortical hyperexcitability is a main pathophysiological mechanism in ALS but different phenotypes could exhibit distinct cortical vulnerability. Also, cortical hyperexcitability exhibits a focal and asymmetrical profile^35^ following a non-random spread pattern, suggesting a focal pathological process with anatomically contiguous extension of motoneuronal degeneration^36^.

From a diagnostic perspective, the presence of the described TMS changes has been reported to result in the reassignment of 88% of Awaji possible into probable or definite, reinforcing the utility of this neurophysiological technique in achieving an earlier diagnosis^26^. Recently, by using SICI in combination with other clinical and electrophysiological variables, an ALS diagnostic index (ALSDI) was developed. The index reliably differentiated this disease from neuromuscular mimicking disorders (area under the curve 0.92, 95% confidence interval (0.89–0.95), with an ALSDI≥4 exhibiting 81.6% sensitivity, 89.6% specificity, and 83.5% diagnostic accuracy^37^. Further studies are needed to evaluate how the diagnostic process is improved by combining this index with the Gold Coast Criteria. Overall, cortical hyperexcitability is an important diagnostic biomarker of ALS, and could enable definitive diagnosis at an earlier stage of the disease.

Only one study^38^ has investigated neuroplastic properties using TMS. It examined the effect of PAS and found that ALS patients exhibit greater LTP-like plasticity over time compared to controls. This effect was nullified by riluzole administration, an antiepileptic drug that blocks glutamate transmission primarily mediated by N-methyl-D-aspartate (NMDA) receptors. These findings suggest that sensorimotor integration in ALS patients is mediated by glutamatergic mechanisms. Additionally, in three patients with focal onset, a facilitated LTP-like response was observed in the hemisphere contralateral to the affected side, which was later replicated in the contralateral hemisphere when symptoms became bilateral. As for rTMS, no studies have been published to date evaluating its diagnostic role in ALS patients.

### Dementias

The clinical syndrome of dementia is associated with several etiologies that lead to neuronal loss and cerebral atrophy. The most common cause of dementia is AD, which accounts for 70% of cases^39^. For its part, FTD is the second most frequent cause in patients under 65 years of age^39^. In this article, we will devote special attention to these dementias with emphasis on the advantages offered by TMS in their differential diagnosis, since they have different patterns of cortical involvement.

#### Alzheimer's disease

AD is a slowly progressive neurodegenerative disease where accumulation of abnormally folded ß-amyloid and tau proteins led to death of neuronal cells, usually beginning in the hippocampal cortex. The pathological process starts decades before symptom onset and hippocampal structural alterations can be observed in the preclinical period^39^.

To date, most TMS studies indicate an increase in cortical excitability in these patients, with some recent research suggesting an inverse association between excitability and cognition in these patients^40^. Exacerbated excitability has been reflected in both increased MEP amplitude^40^ and decreased RMT in M1^41^. It has been seen that hyperexcitability may worsen with disease progression^42^. CSP duration is decreased, indicating a deficient inhibitory control that may promote hyperexcitability^43^ while CMCT remains normal^43^.

Motor cortex investigation using paired-pulse paradigms reveals consistent reduction of SICI, correlating with symptom duration, indicating impaired GABAergic neurotransmission in ALS patients dependent on disease chronicity^41^. GABA inhibits dopamine and acetylcholine synaptic transmission, and dopaminergic agonists^44,45^ and acetylcholinesterase inhibitors^46^ have been shown to restore SICI in Alzheimer patients, suggesting the involvement of GABAA receptors modulated by dopamine and acetylcholine neuromodulators. Comparisons of ICF in Alzheimer patients did not yield significant differences compared to subjects without pathology^41^.

LICI is also decreased in AD^47^, which makes sense if contrasted with the finding of the reduced duration of CSP (mentioned previously) also observed in this group of patients.

**Figure 2:**
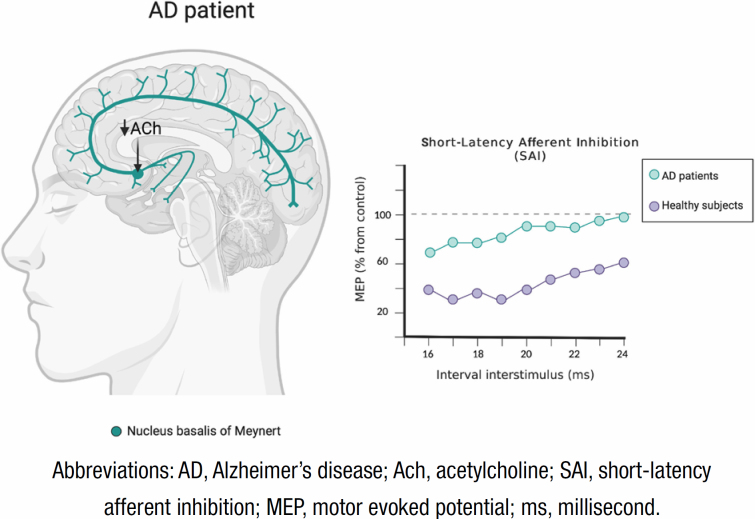
On the left, a schematic of cholinergic innervation from the nucleus basalis of Meynert is shown. In Alzheimer's disease patients, there would be a dysfunction of this nucleus, which would lead to a decrease in the transmission of acetylcholine to the cerebral cortex. And on the right, the result of a cortical excitability protocol, short-latency afferent inhibition, comparing healthy subjects *versus* Alzheimer's disease patients is outlined. Short-latency afferent inhibition is related to the evaluation of cholinergic function at cortical level, so a dysfunction in Meynert’ s nucleus, as in Alzheimer's disease, could cause alterations in this protocol, seen as a decrease in the inhibitory effect in these patients (created with BioRender.com).

More importantly, a consistent reduction in SAI has been seen in these patients^41^. The decrease in this parameter could imply a cholinergic system dysfunction, supported by the pharmacological effect of acetylcholinesterase inhibitors^48^ and by altered Nucleus Basalis of Meynert's connectivity across AD patients spectrum, the main cholinergic nucleus of the basal forebrain^49^ (Figure 2). SAI can be restored by the use of these latter drugs, as well as levodopa, rotigotine, and D2 agonists^44,50^, and even neurostimulation techniques^51^. Other considerations to take into account regarding SAI are that the decrease in this parameter is not necessarily accompanied by an equivalent decrease in cognitive function, and that^52^, furthermore, SAI also decreases with physiological aging^53^.

A clear decrease in neuroplasticity has been observed in studies evaluating PAS^21^. The mechanism by which the PAS protocol is capable of inducing neuroplasticity is through the activation of NMDA-type glutamatergic receptors. Given that no alterations in ICF have been found in Alzheimer patients, this could reflect a preferential alteration in glutamatergic long-term function (LTP) associated with NMDA over short-term glutamatergic transmission (as estimated by ICF).

#### Frontotemporal dementia

FTD is a general term for a group of diverse brain disorders that primarily affect the frontal and temporal lobes of the brain, resulting in progressive dysfunction in executive functioning, behavior, and language^54^. It is classified according to its clinical presentations into behavioral variant (bvFTD) and two forms of primary progressive aphasia (PPA): the non-fluent (nfvPPA) and semantic (svPPA) variants^54^.

In addition, this disease can be also associated with ALS and extrapyramidal syndromes^55^. Up to 12.5%^55^ patients with concomitant ALS diagnosis have been reported. In addition, 27.3% of cases have been reported to present with signs of mild motor dysfunction, such as fatigue and fasciculations^55^. There has therefore been a growing interest in describing neurophysiological biomarkers of motor function in FTD^55^.

Studies with single-pulse TMS have shown dysfunction of the corticospinal tract, reflected in a decreased MEP amplitude^48^ and longer MEP and CMCT latency^55,56^. As mentioned in the previous section, the study of cortical excitability is also a potential tool for differential diagnosis between dementias, which may be challenging in atypical forms of AD presentations, especially for young-onset such as behavioral/dysexecutive variant. Therefore, Alzheimer patients show significantly lower RMT when compared to FTD^57^ and bvFTD^58^. Interestingly, CSP is similar between them, but as a FTD group, CSP is decreased while CMCT is prolonged^56^, suggesting less excitability in the corticomotoneuronal system.

The reduction in SICI/ICF and LICI has been consistently evidenced in the literature. When compared to other neurodegenerative diseases, these patients show reduced SICI than healthy subjects, with no differences seen between FTD (with or without ALS) and pure ALS^55^. Interestingly, this finding appears to be subtype specific despite common pathology, with the reduction in SICI seen particularly in nfvPPA patients but remaining normal in the other variants^55^.

When comparing pre-symptomatic carriers of a pathogenic variant linked to FTD to symptomatic carriers and healthy controls, SICI is only decreased in symptomatic carriers compared to controls, while ICF is reduced both in pre-symptomatic and asymptomatic carriers. The latter may suggest a compromised glutamatergic circuit as an early pathophysiological feature in this group of patients^59^.

SAI is preserved in these patients, supporting preservation of cholinergic function in these patients^60^. Moreover, SAI evaluation is found to be normal when compared to healthy subjects^48^.

In comparison to AD, the use of TMS highlights fundamental differences to FTD, with distinctive profiles of cortical excitability seen for each. The former is characterized by a specific alteration of SAI, while the second demonstrates marked dysfunction in SICI and ICF, respectively. Studies have reported that TMS may differentiate these diseases with 91.8% sensitivity and 88.6% specificity, AD from healthy controls with 84.8% sensitivity and 90.6% specificity, and FTD from healthy subjects with a sensitivity of 90.2% and a specificity of 78.1%^47^.

Regarding plasticity, LTP induced by PAS protocol is impaired in both asymptomatic carriers and patients with pathogenic mutations for FTD, possibly representing an early biomarker of neurodegeneration^59^.

Di Stasio et al. studied these patients with and without parkinsonian symptoms using TBS. Patients presenting with parkinsonism had an abnormal response to TBS, but the response was normal in patients without it. Furthermore, there was a similar response to TBS between FTD patients with parkinsonism and patients with PD, implying neurodegeneration in corticobasal ganglia-thalamocortical motor networks^61^. The change in LTP induced by iTBS after treatment with a neuroprotective endocannabinoid in patients with FTD have been used to assess neuroplasticity and, thus, could be used as a theranostic biomarker in the future^62^.

### Parkinson's disease

PD is a neurodegenerative disorder affecting 2-4% of individuals over 85 years of age^63^ that affects several neural networks, leading to a broad spectrum of motor and extra motor symptoms that impair function and quality of life^63^. The disease also encompasses various nonmotor symptoms, including cognitive deficits^64^. Currently, there is no known curative treatment. Therefore, a comprehensive understanding of its pathophysiology is essential.

In general terms, PD has increased corticospinal excitability compared to the control group^65^ and a shortened CSP^66^. However, in terms of cortical excitability using paired- pulse protocols, PD studies show conflicting results. Some researchers report normality in parameters such as SICI and ICF^67^, with results not being reproduced by other groups^68^.

Different patterns of alterations in motor cortical excitability have been observed in PD, showed as a decrease in ICF in cortical lower limb representation related to gait hypokinesia^69^, and SICI impairment in upper limb cortical areas^70^, suggesting different alterations in intracortical circuits in M1. Interestingly, one motor complication of PD, levodopa induced dyskinesias, has been correlated with decreased SICI and LICI along with an increased ICF and SICF^71^, suggesting that non- dopaminergic pathways contribute to the development of this complication.

SAI studies have also shown variable results^72,73^. Interestingly, there has been a significant reduction of SAI in patients with PD and concomitant dementia compared to Parkinson patients without cognitive dysfunction^74^. The degree of SAI impairment seems to be comparable to that in AD and shows similar correlation with cognitive dysfunction. Also, reduction of SAI has been associated with non-motor symptoms such as REM sleep behavior disorder, visual hallucinations, olfactory impairment, and dysphagia^73^.

The excitability profile of various gene mutations in PD has also been explored using TMS. Patients with leucine-rich repeat kinase 2 (LRRK2) gene mutations show reduced SICI and an increase in ICF in contrast to the idiopathic disease group^75,76^, and *Parkin* and *PINK1* mutations carriers exhibit hyperexcitable premotor-M1 connectivity using twin-coil TMS^77^, suggesting a disruption of the normal excitatory inhibitory intracortical balance underlying the phenotypic similarity of these patients. Also, CBI is altered in PD patients, which suggests a dysfunction in cerebellar-thalamocortical projections^78^. This was particularly seen in patients with evidence of a dopaminergic deficit on imaging, indicating that such impairments in CBI may be a biomarker for dopamine deficiency^79^.

While TMS has not yet been utilized as a predictive tool for cognitive decline in PD, the significant consequences of cognitive impairment in this population warrant further investigation. The potential of TMS as a biomarker or predictive measure holds promise for improving patient outcomes and enhancing our understanding of cognitive dysfunction in PD.

Studies of cortical plasticity in PD have remained controversial. PAS-induced plasticity has been reported to be reduced compared to healthy controls^80^, while other researchers have shown normal plasticity in these patients^81^. LTP-like plasticity generated by iTBS is impaired in patients, regardless of medication status or levodopa- induced dyskinesias^82^. On the other hand, in studies in which an alteration in PAS was observed, levodopa administration was able to restore this parameter in non-dyskinetic but not in dyskinetic patients, suggesting that abnormal synaptic plasticity in M1 could be important for the development of levodopa- induced dyskinesias^82^.

In conclusion, TMS techniques hold significant promise in aiding the diagnosis of various neurodegenerative diseases and the differentiation between their subtypes. However, it is crucial to approach data interpretation with caution due to inconsistencies observed across studies. These inconsistencies may arise from inadequate cohort sizes and the considerable heterogeneity in the clinical presentation and severity of the diseases under investigation. Therefore, addressing these issues represents an opportunity to enhance the internal validation of these studies.

In addition to these challenges, it i s important to recognize that most of the TMS paradigms, while highly valuable for assessing cortical properties such as excitability, plasticity, and connectivity, have inherent technical limitations. Firstly, these paradigms are primarily limited to exploring the motor system and cannot be easily extended to non-motor regions, primarily because they rely on the motor response. Secondly, MEP, a pivotal measure in this setup, is influenced not only by cortical mechanisms but also by factors related to spinal excitability and muscle properties. One potential solution to address these limitations is the utilization of TMS-EEG techniques, which do not require muscular effectors and can offer insights into cortical activity with greater applicability across diverse brain regions^13^. Despite these pending challenges and technical limitations, TMS techniques still hold the potential to contribute significantly to the clinical diagnosis of neurodegenerative diseases by shedding light on diverse pathophysiological aspects of these conditions in a safe and non-invasive manner.

Lastly, it is worth noting that the majority of neurophysiological studies utilizing TMS have predominantly focused on Caucasian or Asian populations. Therefore, there is an urgent need to develop and promote the utilization of these techniques in LAC countries to enhance the global validity of the results. Furthermore, exploring whether the phenotypic variability of neurodegenerative diseases leads to differences in neurophysiological characterization using TMS techniques is an intriguing avenue for research. Consequently, we recommend fostering collaborative partnerships to initiate multicenter studies that encompass the diverse LAC population.

## References

[1] Rachakonda V, Pan TH, Le WD (2004). Biomarkers of neurodegenerative disorders: how good are they?. Cell Res.

[2] Chertkow H, Feldman HH, Jacova C, Massoud F (2013). Definitions of dementia and predementia states in Alzheimer's disease and vascular cognitive impairment: consensus from the Canadian conference on diagnosis of dementia. Alzheimers Res Ther.

[3] Román GC (1996). Neuroepidemiology of amyotrophic lateral sclerosis: clues to aetiology and pathogenesis. J Neurol Neurosurg Psychiatry.

[4] United Nations. Department of Economic and Social Affairs. Population Division (2020). World Population Ageing 2020 Highlights: Living Arrangements of Older Persons.

[5] Zheng JC, Chen S (2022). Translational Neurodegeneration in the era of fast growing international brain research. Transl Neurodegener.

[6] Ou Z, Pan J, Tang S, Duan D, Yu D, Nong H (2021). Global Trends in the Incidence, Prevalence, and Years Lived With Disability of Parkinson's Disease in 204 Countries/Territories From 1990 to 2019. Front Public Health.

[7] Hansson O (2021). Biomarkers for neurodegenerative diseases. Nat Med.

[8] Duran-Aniotz C, Orellana P, Leon Rodriguez T, Henriquez F, Cabello V, Aguirre-Pinto MF (2021). Systematic Review: Genetic, Neuroimaging, and Fluids Biomarkers for Frontotemporal Dementia Across Latin America Countries. Front Neurol.

[9] Merton PA, Morton HB (1980). Stimulation of the cerebral cortex in the intact human subject. Nature.

[10] Barker AT, Jalinous R, Freeston IL (1985). Non-Invasive Magnetic Stimulation of Human Motor Cortex. Lancet.

[11] Di Lazzaro V, Oliviero A, Profice P, Saturno E, Pilato F, Insola A (1998). Comparison of descending volleys evoked by transcranial magnetic and electric stimulation in conscious humans. Electroencephalogr Clin Neurophysiol.

[12] Connolly KR, Helmer A, Cristancho MA, Cristancho P, O’Reardon JP (2012). Effectiveness of transcranial magnetic stimulation in clinical practice post-FDA approval in the United States: results observed with the first 100 consecutive cases of depression at an academic medical center. J Clin Psychiatry.

[13] Tremblay S, Rogasch NC, Premoli I, Blumberger DM, Casarotto S, Chen R (2019). Clinical utility and prospective of TMS-EEG. Clin Neurophysiol.

[14] Rossini PM, Burke D, Chen R, Cohen LG, Daskalakis Z, Di Iorio R (2015). Non-invasive electrical and magnetic stimulation of the brain, spinal cord, roots and peripheral nerves: Basic principles and procedures for routine clinical and research application. An updated report from an I.F.C.N. Committee. Clin Neurophysiol.

[15] Tokimura H, Ridding MC, Tokimura Y, Amassian VE, Rothwell JC (1996). Short latency facilitation between pairs of threshold magnetic stimuli applied to human motor cortex. Electroencephalogr Clin Neurophysiol.

[16] Di Lazzaro V, Bella R, Benussi A, Bologna M, Borroni B, Capone F (2021). Diagnostic contribution and therapeutic perspectives of transcranial magnetic stimulation in dementia. Clin Neurophysiol.

[17] Fisher RJ, Nakamura Y, Bestmann S, Rothwell JC, Bostock H (2002). Two phases of intracortical inhibition revealed by transcranial magnetic threshold tracking. Exp Brain Res.

[18] Vucic S, Howells J, Trevillion L, Kiernan MC (2006). Assessment of cortical excitability using threshold tracking techniques. Muscle Nerve.

[19] Samusyte G, Bostock H, Rothwell J, Koltzenburg M (2017). P157 Reliability of threshold tracking technique for short interval intracortical inhibition. Clin Neurophysiol.

[20] Colom-Cadena M, Spires-Jones T, Zetterberg H, Blennow K, Caggiano A, DeKosky ST (2020). The clinical promise of biomarkers of synapse damage or loss in Alzheimer's disease. Alzheimers Res Ther.

[21] Battaglia F, Wang HY, Ghilardi MF, Gashi E, Quartarone A, Friedman E (2007). Cortical plasticity in Alzheimer's disease in humans and rodents. Biol Psychiatry.

[22] Suppa A, Quartarone A, Siebner H, Chen R, Di Lazzaro V, Del Giudice P (2017). The associative brain at work: Evidence from paired associative stimulation studies in humans. Clin Neurophysiol.

[23] Al-Chalabi A, Calvo A, Chio A, Colville S, Ellis CM, Hardiman O (2014). Analysis of amyotrophic lateral sclerosis as a multistep process: a population-based modelling study. Lancet Neurol.

[24] Shefner JM, Al-Chalabi A, Baker MR, Cui LY, de Carvalho M, Eisen A (2020). A proposal for new diagnostic criteria for ALS. Clin Neurophysiol.

[25] Swash M (2012). Why are upper motor neuron signs difficult to elicit in amyotrophic lateral sclerosis?. J Neurol Neurosurg Psychiatry.

[26] Geevasinga N, Menon P, Yiannikas C, Kiernan MC, Vucic S (2014). Diagnostic utility of cortical excitability studies in amyotrophic lateral sclerosis. Eur J Neurol.

[27] Van den Bos MAJ, Higashihara M, Geevasinga N, Menon P, Kiernan MC, Vucic S (2018). Imbalance of cortical facilitatory and inhibitory circuits underlies hyperexcitability in ALS. Neurology.

[28] Cengiz B, Fidanci H, Keçeli YK, Baltaci H, KuruoĞlu R (2019). Impaired short- and long-latency afferent inhibition in amyotrophic lateral sclerosis. Muscle Nerve.

[29] Higashihara M, Pavey N, van den Bos M, Menon P, Kiernan MC, Vucic S (2021). Association of cortical hyperexcitability and cognitive impairment in patients With amyotrophic lateral sclerosis. Neurology.

[30] Zhang W, Zhang L, Liang B, Schroeder D, Zhang ZW, Cox GA (2016). Hyperactive somatostatin interneurons contribute to excitotoxicity in neurodegenerative disorders. Nat Neurosci.

[31] Khademullah CS, Aqrabawi AJ, Place KM, Dargaei Z, Liang X, Pressey JC (2020). Cortical interneuron-mediated inhibition delays the onset of amyotrophic lateral sclerosis. Brain.

[32] Vucic S, Nicholson GA, Kiernan MC (2008). Cortical hyperexcitability may precede the onset of familial amyotrophic lateral sclerosis. Brain.

[33] Geevasinga N, Menon P, Nicholson GA, Ng K, Howells J, Kril JJ (2015). Cortical Function in Asymptomatic Carriers and Patients With C9orf72 Amyotrophic Lateral Sclerosis. JAMA Neurol.

[34] Turner MR (2005). Abnormal cortical excitability in sporadic but not homozygous D90A SOD1 ALS. J Neurol Neurosurg Psychiatry.

[35] Menon P, Geevasinga N, van den Bos M, Yiannikas C, Kiernan MC, Vucic S (2017). Cortical hyperexcitability and disease spread in amyotrophic lateral sclerosis. Eur J Neurol.

[36] Ravits JM, La Spada AR (2009). ALS motor phenotype heterogeneity, focality, and spread: Deconstructing motor neuron degeneration. Neurology.

[37] Geevasinga N, Howells J, Menon P, van den Bos M, Shibuya K, Matamala JM (2019). Amyotrophic lateral sclerosis diagnostic index: Toward a personalized diagnosis of ALS. Neurology.

[38] Ceccanti M, Onesti E, Rubino A, Cambieri C, Tartaglia G, Miscioscia A (2018). Modulation of human corticospinal excitability by paired associative stimulation in patients with amyotrophic lateral sclerosis and effects of Riluzole. Brain Stimul.

[39] Erkkinen MG, Kim MO, Geschwind MD (2018). Clinical Neurology and Epidemiology of the Major Neurodegenerative Diseases. Cold Spring Harb Perspect Biol.

[40] Joseph S, Patterson R, Wang W, Blumberger DM, Rajji T, Kumar S (2022). Quantitative Assessment of Cortical Excitability in Alzheimer's Dementia and Its Association with Clinical Symptoms: A Systematic Review and Meta-Analyses. J Alzheimers Dis.

[41] Mimura Y, Nishida H, Nakajima S, Tsugawa S, Morita S, Yoshida K (2021). Neurophysiological biomarkers using transcranial magnetic stimulation in Alzheimer's disease and mild cognitive impairment: A systematic review and meta-analysis. Neurosci Biobehav Rev.

[42] Zadey S, Buss SS, McDonald K, Press DZ, Pascual-Leone A, Fried PJ (2021). Higher motor cortical excitability linked to greater cognitive dysfunction in Alzheimer's disease: results from two independent cohorts. Neurobiol Aging.

[43] Perretti A, Grossi D, Fragassi N, Lanzillo B, Nolano M, Pisacreta AI (1996). Evaluation of the motor cortex by magnetic stimulation in patients with Alzheimer disease. J Neurol Sci.

[44] Martorana A, Mori F, Esposito Z, Kusayanagi H, Monteleone F, Codecà C (2009). Dopamine modulates cholinergic cortical excitability in Alzheimer's disease patients. Neuropsychopharmacology.

[45] Nardone R, Höller Y, Thomschewski A, Kunz AB, Lochner P, Golaszewski S (2014). Dopamine differently modulates central cholinergic circuits in patients with Alzheimer disease and CADASIL. J Neural Transm.

[46] Pierantozzi M, Panella M, Palmieri MG, Koch G, Giordano A, Marciani MG (2004). Different TMS patterns of intracortical inhibition in early onset Alzheimer dementia and frontotemporal dementia. Clin Neurophysiol.

[47] Benussi A, Di Lorenzo F, Dell’Era V, Cosseddu M, Alberici A, Caratozzolo S (2017). Transcranial magnetic stimulation distinguishes Alzheimer disease from frontotemporal dementia. Neurology.

[48] Di Lazzaro V, Pilato F, Dileone M, Saturno E, Oliviero A, Marra C (2006). In vivo cholinergic circuit evaluation in frontotemporal and Alzheimer dementias. Neurology.

[49] Liu AKL, Chang RCC, Pearce RKB, Gentleman SM (2015). Nucleus basalis of Meynert revisited: anatomy, history and differential involvement in Alzheimer's and Parkinson's disease. Acta Neuropathol.

[50] Martorana A, Di Lorenzo F, Esposito Z, Lo Giudice T, Bernardi G, Caltagirone C (2013). Dopamine D_2_-agonist rotigotine effects on cortical excitability and central cholinergic transmission in Alzheimer's disease patients. Neuropharmacology.

[51] Di Lorenzo F, Martorana A, Ponzo V, Bonnì S, D’Angelo E, Caltagirone C (2013). Cerebellar theta burst stimulation modulates short latency afferent inhibition in Alzheimer's disease patients. Front Aging Neurosci.

[52] Di Lorenzo F, Ponzo V, Bonnì S, Motta C, Negrão Serra PC, Bozzali M (2016). Long-term potentiation-like cortical plasticity is disrupted in Alzheimer's disease patients independently from age of onset. Ann Neurol.

[53] Young-Bernier M, Kamil Y, Tremblay F, Davidson PSR (2012). Associations between a neurophysiological marker of central cholinergic activity and cognitive functions in young and older adults. Behav Brain Funct.

[54] Burrell JR, Halliday GM, Kril JJ, Ittner LM, Götz J, Kiernan MC (2016). The frontotemporal dementia-motor neuron disease continuum. Lancet.

[55] Burrell JR, Kiernan MC, Vucic S, Hodges JR (2011). Motor neuron dysfunction in frontotemporal dementia. Brain.

[56] Chandra SR, Issac TG, Nagaraju BC, Philip M (2016). A Study of Cortical Excitability, Central Motor Conduction, and Cortical Inhibition Using Single Pulse Transcranial Magnetic Stimulation in Patients with Early Frontotemporal and Alzheimer's Dementia. Indian J Psychol Med.

[57] Issac TG, Chandra SR, Nagaraju BC (2013). Transcranial magnetic stimulation in patients with early cortical dementia: A pilot study. Ann Indian Acad Neurol.

[58] Wang P, Zhang H, Han L, Zhou Y (2016). Cortical function in Alzheimer's disease and frontotemporal dementia. Transl Neurosci.

[59] Benussi A, Cosseddu M, Filareto I, Dell’Era V, Archetti S, Sofia Cotelli M (2016). Impaired long-term potentiation-like cortical plasticity in presymptomatic genetic frontotemporal dementia. Ann Neurol.

[60] Benussi A, Dell’Era V, Cantoni V, Cotelli MS, Cosseddu M, Spallazzi M (2020). TMS for staging and predicting functional decline in frontotemporal dementia. Brain Stimul.

[61] Di Stasio F, Suppa A, Fabbrini A, Marsili L, Asci F, Conte A (2018). Parkinsonism is associated with altered primary motor cortex plasticity in frontotemporal dementia-primary progressive aphasia variant. Neurobiol Aging.

[62] Assogna M, Casula EP, Borghi I, Bonnì S, Samà D, Motta C (2020). Effects of Palmitoylethanolamide Combined with Luteoline on Frontal Lobe Functions, High Frequency Oscillations, and GABAergic Transmission in Patients with Frontotemporal Dementia. J Alzheimers Dis.

[63] Simon DK, Tanner CM, Brundin P (2020). Parkinson Disease Epidemiology, Pathology, Genetics, and Pathophysiology. Clin Geriatr Med.

[64] Fang C, Lv L, Mao S, Dong H, Liu B (2020). Cognition Deficits in Parkinson's Disease: Mechanisms and Treatment. Parkinsons Dis.

[65] Ni Z, Bahl N, Gunraj CA, Mazzella F, Chen R (2013). Increased motor cortical facilitation and decreased inhibition in Parkinson disease. Neurology.

[66] Cantello R, Gianelli M, Bettucci D, Civardi C, De Angelis MS, Mutani R (1991). Parkinson's disease rigidity: magnetic motor evoked potentials in a small hand muscle. Neurology.

[67] Chu J, Wagle-Shukla A, Gunraj C, Lang AE, Chen R (2009). Impaired presynaptic inhibition in the motor cortex in Parkinson disease. Neurology.

[68] Bares M, Kanovský P, Klajblová H, Rektor I (2003). Intracortical inhibition and facilitation are impaired in patients with early Parkinson's disease: a paired TMS study. Eur J Neurol.

[69] Vacherot F, Attarian S, Eusebio A, Azulay JP (2010). Excitability of the lower-limb area of the motor cortex in Parkinson's disease. Neurophysiol Clin.

[70] Cantello R, Tarletti R, Civardi C (2002). Transcranial magnetic stimulation and Parkinson's disease. Brain Res Brain Res Rev.

[71] Guerra A, Suppa A, D’Onofrio V, Di Stasio F, Asci F, Fabbrini G (2019). Abnormal cortical facilitation and L-dopa-induced dyskinesia in Parkinson's disease. Brain Stimul.

[72] Sailer A, Molnar GF, Paradiso G, Gunraj CA, Lang AE, Chen R (2003). Short and long latency afferent inhibition in Parkinson's disease. Brain.

[73] Manganelli F, Vitale C, Santangelo G, Pisciotta C, Iodice R, Cozzolino A (2009). Functional involvement of central cholinergic circuits and visual hallucinations in Parkinson's disease. Brain.

[74] Celebi O, Temuçin CM, Elibol B, Saka E (2012). Short latency afferent inhibition in Parkinson's disease patients with dementia. Mov Disord.

[75] Kojovic M, Kassavetis P, Pareés I, Georgiev D, Rocchi L, Balint B (2017). Pathophysiological heterogeneity in Parkinson's disease: Neurophysiological insights from LRRK2 mutations. Mov Disord.

[76] Ponzo V, Di Lorenzo F, Brusa L, Schirinzi T, Battistini S, Ricci C (2017). Impaired intracortical transmission in G2019S leucine rich-repeat kinase Parkinson patients. Mov Disord.

[77] Weissbach A, Bäumer T, Pramstaller PP, Brüggemann N, Tadic V, Chen R (2017). Abnormal premotor-motor interaction in heterozygous Parkin- and Pink1 mutation carriers. Clin Neurophysiol.

[78] Carrillo F, Palomar FJ, Conde V, Diaz-Corrales FJ, Porcacchia P, Fernández-Del-Olmo M (2013). Study of cerebello-thalamocortical pathway by transcranial magnetic stimulation in Parkinson's disease. Brain Stimul.

[79] Schirinzi T, Di Lorenzo F, Ponzo V, Palmieri MG, Bentivoglio AR, Schillaci O (2016). Mild cerebello-thalamo-cortical impairment in patients with normal dopaminergic scans (SWEDD). Parkinsonism Relat Disord.

[80] Lu MK, Chen CM, Duann JR, Ziemann U, Chen JC, Chiou SM (2016). Investigation of Motor Cortical Plasticity and Corticospinal Tract Diffusion Tensor Imaging in Patients with Parkinsons Disease and Essential Tremor. PLoS One.

[81] Zamir O, Gunraj C, Ni Z, Mazzella F, Chen R (2012). Effects of theta burst stimulation on motor cortex excitability in Parkinson's disease. Clin Neurophysiol.

[82] Suppa A, Marsili L, Belvisi D, Conte A, Iezzi E, Modugno N (2011). Lack of LTP-like plasticity in primary motor cortex in Parkinson's disease. Exp Neurol.

